# Combined Effect of Textured Patterns and Graphene Flake Additives on Tribological Behavior under Boundary Lubrication

**DOI:** 10.1371/journal.pone.0152143

**Published:** 2016-04-07

**Authors:** Zhen-bing Cai, Lei Zhao, Xu Zhang, Wen Yue, Min-hao Zhu

**Affiliations:** 1 Key Lab of Advanced Technologies of Materials (Ministry of Education), Tribology Research Institute, Southwest Jiaotong University, Chengdu, 610031, China; 2 Mechanical Engineering Department, School of Engineering and Technology, China University of Geosciences (Beijing), 100083, China; SPECS Surface Nano Analysis GmbH, GERMANY

## Abstract

A ball-on-plate wear test was employed to investigate the effectiveness of graphene (GP) nanoparticles dispersed in a synthetic-oil-based lubricant in reducing wear. The effect by area ratio of elliptically shaped dimple textures and elevated temperatures were also explored. Pure PAO4 based oil and a mixture of this oil with 0.01 wt% GP were compared as lubricants. At pit area ratio of 5%, GP-base oil effectively reduced friction and wear, especially at 60 and 100°C. Under pure PAO4 oil lubrication, the untextured surfaces gained low friction coefficients (COFs) and wear rates under 60 and 100°C. With increasing laser—texture area ratio, the COF and wear rate decreased at 25 and 150°C but increased at 60 and 100°C. Under the GP-based oil lubrication, the textured surface with 5% area ratio achieved the lowest COF among those of the area ratios tested at all test temperatures. Meanwhile, the textured surface with 20% area ratio obtained the highest COF among those of the area ratios. With the joint action of GP and texture, the textured surface with 10% area ratio exhibited the best anti-wear performance among all of the textured surfaces at all test temperatures.

## Introduction

Graphene (GP) is a monolayer of graphite (2D) and is the basic building block of all graphitic forms, such as fullerenes (0D), carbon nanotubes (1D), and graphite (3D) [1]. GP possesses numerous excellent properties, such as extreme thinness, high mechanical strength, high electrical conductivity, high surface area, and thermal mechanical properties. Thus, GP has been attracting worldwide interest since its discovery [2–4]. These unique properties render GP a promising candidate for different applications, such as in transistor—transparent electrodes, chemical and biological sensors, and energy-storage materials.

However, only a few studies on the tribological applications of GP have been reported, particularly, the addition of nano-GP particles into lubricating oil [5]. GP easily aggregates in solution and difficultly forms a stable dispersion, thereby limiting the application in the field of lubrication. GP exhibits good chemical stability and possesses massive inter-layer van der Waals forces. Hence, forming a stable dispersion solution with GP is difficult to achieve, especially on account of the agglomeration phenomenon [6]. Two kinds of methods are often used to improve the dispersibility of GP in oils. One is adding a dispersant, which leads to a uniform dispersion of GP in oil. Another is modifying the surface appropriately to enhance the lipophilic property of GP. Tadmor [7] and N'guessan’s [8] used the centrifugal adhesion balance method to measurements of the lateral adhesion forces at a solid-liquid interface in GP. Varrla et al. [9] reported that the coefficient of friction (COF) and the wear scar diameter are reduced by 80% and 33%, respectively, when the concentration of GP is 0.025 mg/mL in base oil. Lin et al. [10] found the significant reduction in frictional coefficient and wear using GP modified by stearic/oleic acid as lubricant additive. To improve the effective functioning of two friction surfaces, lubricants with additives are required. However, surface texturing is another efficient approach to improve the interface [11–13]. Through this approach, hydrodynamic lubrication is facilitated, lubricants are stored, and the interface contains the debris [14–17]. To date, most of the research on this aspect has been conducted on smooth surfaces. The combined effect of surface textures and additives on friction interfaces is worth studying and exploring.

In the present work, we prepared oil fluid containing GP and texturized the surface under study by laser. Tribology tests were carried out under different test conditions, and the interaction between the textured surface and additive was investigated.

## Experimental Procedure

### Experimental details

Tribological performance was evaluated on a UTM-2 tribometer with a ball-on-plate configuration, in which the lower plate was immersed in oil. The normal load during the wear test was 5 N, and the sliding speed was 5 mm/s. A boundary lubrication condition could be ensured at such a low sliding speed and even at a light load.

The sliding distance was 8 mm, and the temperatures were 25, 60, 100, and 150°C. Testing lasted for 6000 s. The temperature of the friction surface was measured by a thermocouple installed at the fixed plate, whereas the friction coefficient was measured and recorded as a function of the normal load. The steel ball (GCr15) used was 9.525 mm in diameter, and the bronze surface (brand of 633; composition (wt%): 6.1 Sn, 6.5 Zn, 2.1 Pb, bal-Cu) measured 25 mm × 12 mm × 6 mm.

**Fig 1:**
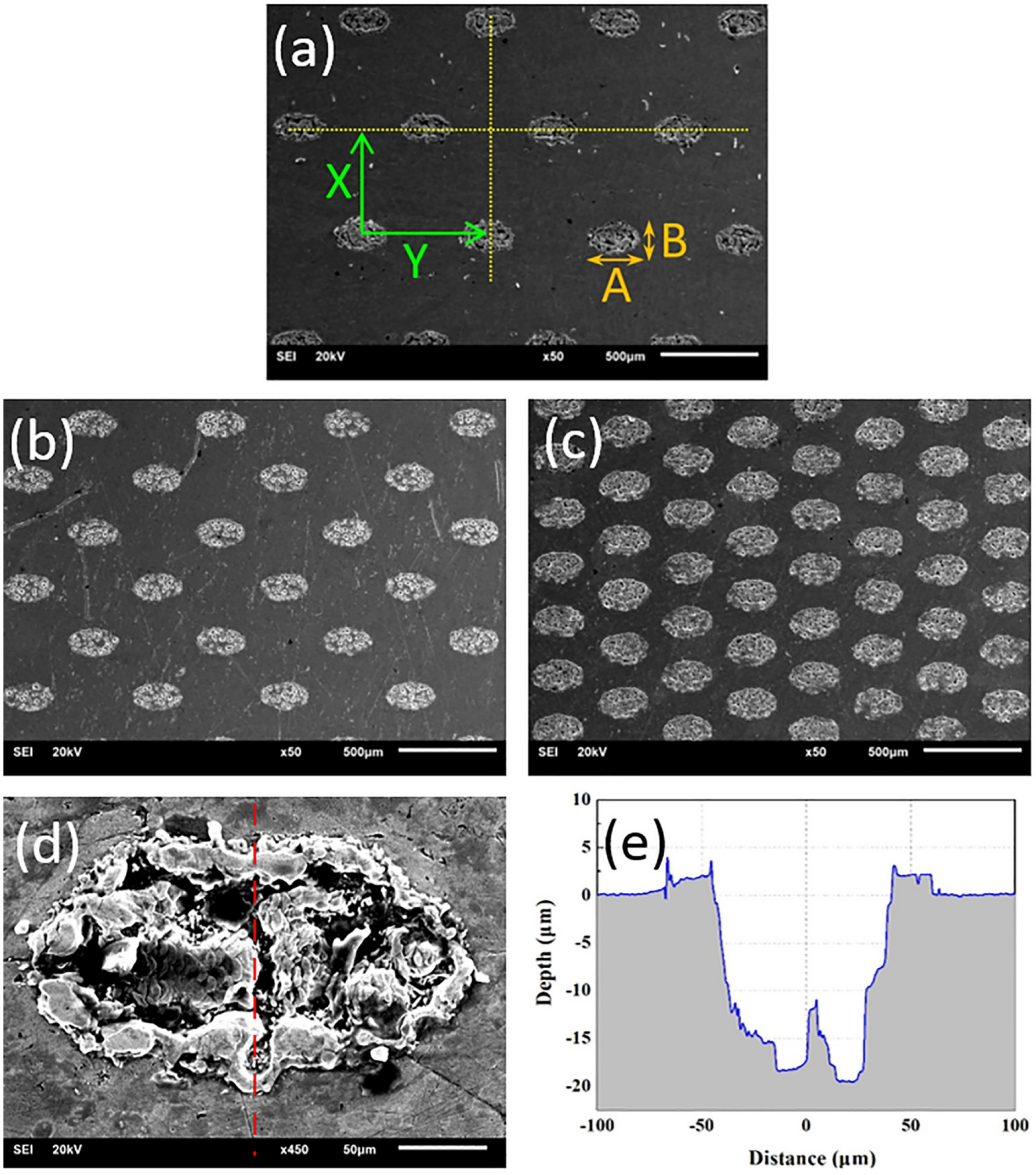
Morphologies and profiles of the laser-textured surfaces, (a) 1#, r = 5%, (b) 2#, r = 10%, (c) 3#, r = 20%(d) Dimple, (e) Profile.

The bronze surface possessed elliptical dimples generated by laser etching (pulse, Nd:YAG Laser Processing System, wavelength = 1064 nm, mean power = 10 W, pulse width = 5–25 ns, processing pulse = 10 kHz, and scan speed = 5 mm/s). Four kinds of plates with differently textured areas denoted as 0#, 1#, 2#, and 3# were used, with the corresponding area ratios of 0%, 5%, 10%, and 20%, respectively. The dimples had Ellipse dimensions(long axis-200μm and short axis-100μm and depth of 20μm). Table 1 indicates the geometric parameters of the textured surfaces. Fig 1 shows the textured surfaces. When the laser beam hit the bronze surface, a temperature gradient was generated on the surface. This temperature gradient induced the formation of a surface-tension gradient toward the molten pool, causing the material to amass at the pool edge.

**Table 1:** Geometric parameters of the textured surfaces.

Parameters (μm)	Sample number			
	0#	1#	2#	3#
Long axis of the dimple (A)	0	200	200	200
Short axis of the dimple (B)	0	100	100	100
Texture horizontal spacing (Y)	0	300	300	300
Texture vertical spacing (X)	0	523.6	261.8	130.9
Depth of dimple	0	20	20	20
Area ratio (r)	0	5%	10%	20%

High-resolution transmission electron microscopy (HR-TEM; JEM-2100F), X-ray diffraction (XRD), Raman spectroscopy, and Fourier transform infrared spectroscopy (FT-IR) were employed to reveal the GP morphologies. Prior to HR-TEM, a droplet of fluid suspension was combined with an ethanol drop on a silicon pellet and was then allowed to stand and dry for 24 h. The powder XRD measurements were performed with an X-pert Pro MPD X-ray diffractometer using nickel-filtered Cu K α (1.54 Å) radiation as the X-ray source. The pattern was recorded in the 2θ range of 0°–60° with a step size of 0.02°. Raman spectra characterization (Lab Ram HR) utilized a 532 nm laser as the excitation source (Via In, Renishaw, Ar, ion laser, scanning range of 500–4000 cm^–1^, wave number accuracy of 1 cm). FT-IR (Nicolet 5700) studies were performed using a Perkin-Elmer spectrum one spectrometer in the range of 400–4000 cm^−1^ using KBr pellets.

### Lubricant and additive dispensability

The tribology pairs were lubricated with a synthetic PAO base oil with 0.01 wt% of commercial nanoparticle GP.

**Fig 2:**
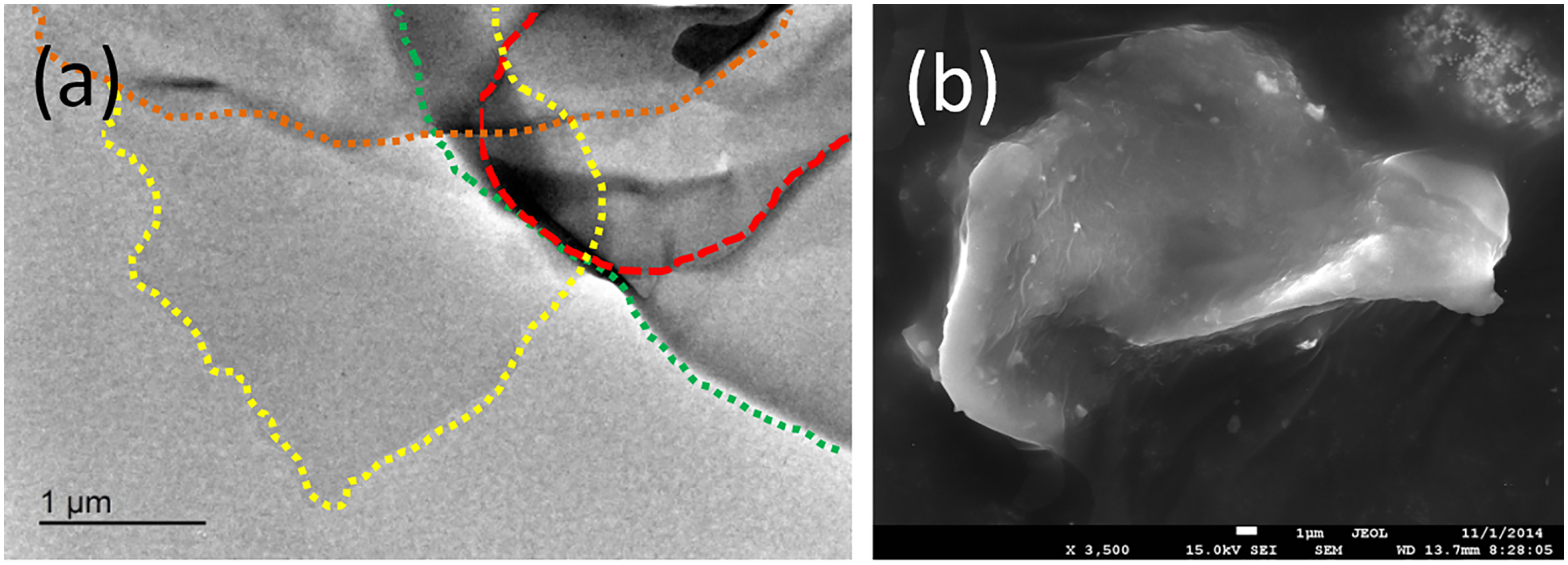
GP particle morphology observed under (a) TEM(The dotted line represents the boundary of the GP) and (b) scanning electron microscopy (SEM).

The GP particles used in this research was purchased from Jinan Graphene New Materials Co. (MX-AVS). Their morphologies and structures are shown in Fig 2. The transmission electron microscopy (TEM) image shown in Fig 2a clearly demonstrates that the GP sample possesses ultra-nanometer dimensions with flat single flakes and wrinkles. Wrinkles are necessary for the 2D GP because the single flakes maintain the surface energy of GP [18].

**Fig 3:**
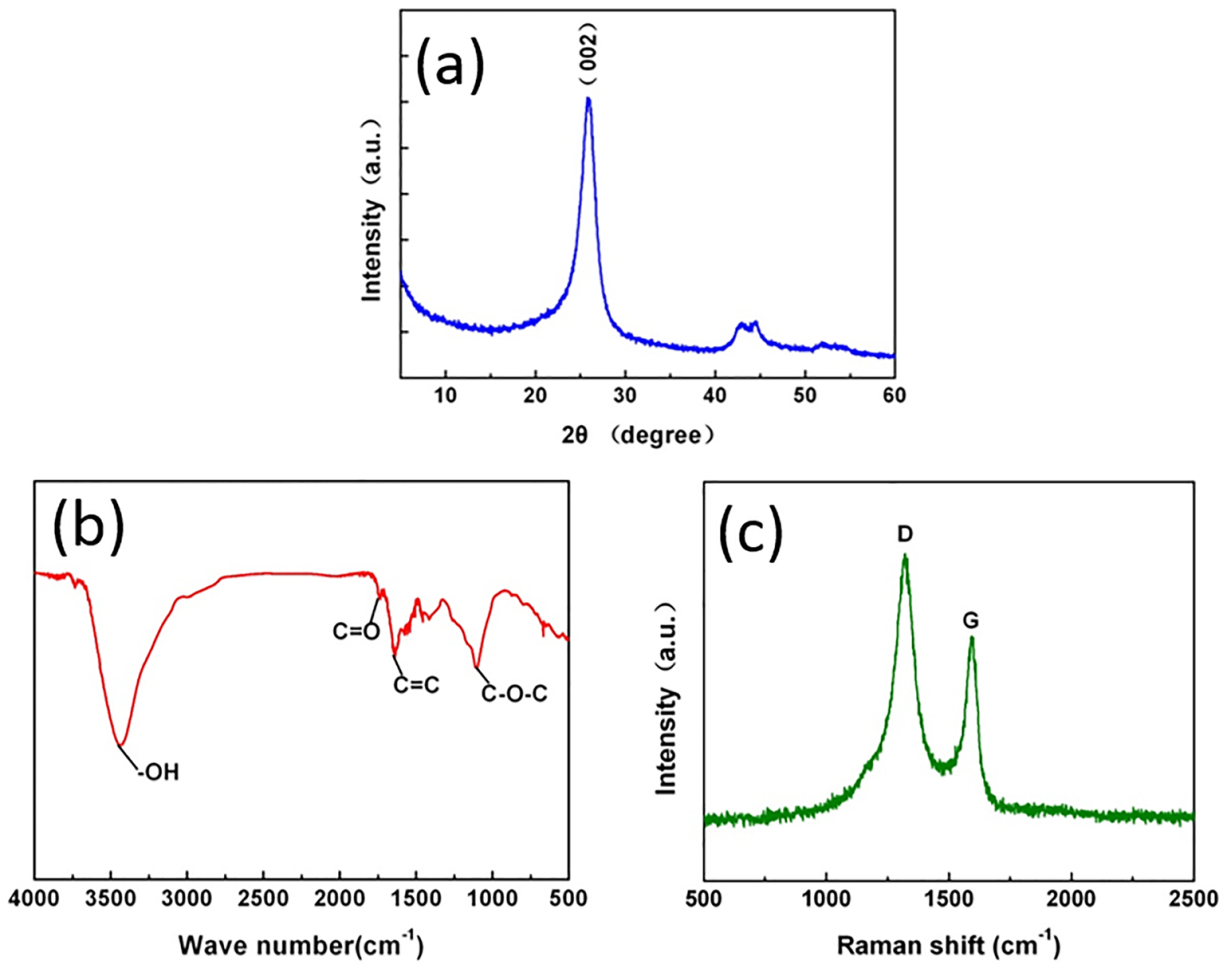
Characterization of the graphene particles, (a) XRD pattern, (b) Infrared spectra, (c) Raman spectrum.

Fig 3a displays the XRD diffractograms of GP conducted to explore the structure and interlayer distance. A diffraction peak is shown at 26° (002), and Bragg’s equation was applied to the (002) reflection to evaluate the distance between GP layers, denoted as 0.34 nm. It is worthy to note that a peaks appear at 44°, and it mean that the presence of a small amount of graphite. From the FT-IR spectrum, Fig 3b shows the FT-IR spectrum of the graphite crystal. The peak at 1106 cm^−1^ implies the absorption of the C—O—C bonds. Meanwhile, the peak at 1637 cm^−1^ corresponds to C = C, particularly the sp^2^ carbon of the GP. The C = O absorption peak is located at 1735 cm^−1^, and the O—H absorption peak wave number varies within 3000–3700 cm^−1^. To obtain additional information on the GP structure, Raman spectroscopy of the GP samples was conducted (Fig 3c). Two peaks, particularly, the D and G bands, are the typical features of GP. The D peak (1320 cm^−1^) corresponds to the sp^3^ hybrid structures (tetrahedron structure) or the sp^2^ hybridized health defects (GP edge structure). By contrast, the G band (1590 cm^−1^) indicates the vibration in all of the sp^2^-bonded carbon atoms, thereby denoting a “defect-free” graphitic character.

**Fig 4:**
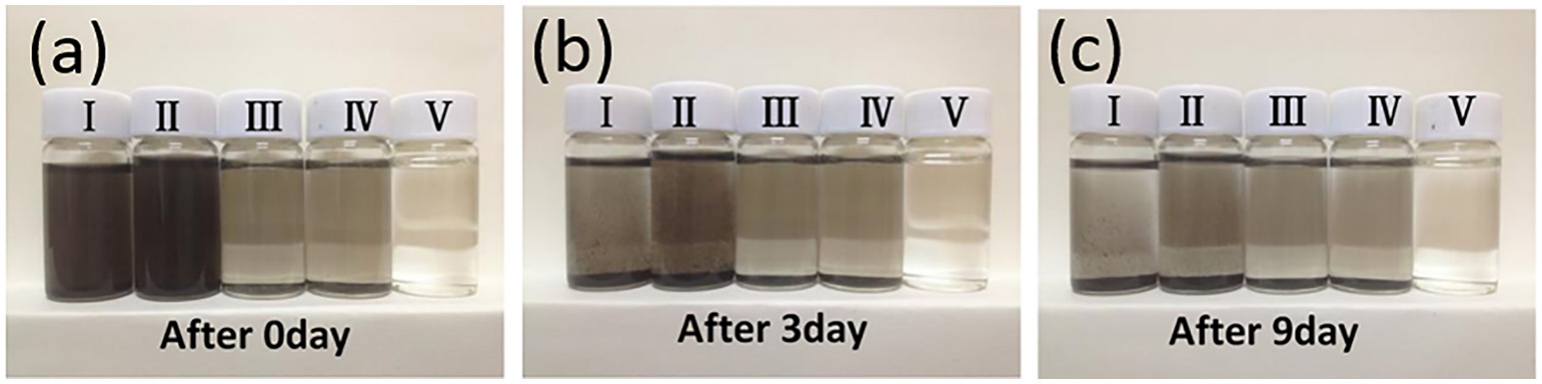
Optical images of GP dispersion in base oil (a) upon addition, (b) after three days, and (c) after nine days(I: PAO4 + 0.05% GP, II: PAO4 + 0.05% GP + 1% SP, III: PAO4 + 0.01% GP, IV: PAO4 + 0.01% GP + 1% SP, and V: PAO4.)

To attain an enhanced GP dispersion, Span-80 (SP, C_24_H_44_O_6_; Kelong Chemical Reagent Factory, Chengdu, China) was selected as dispersant. The specific procedures adopted are as follows. First, 0.01 wt% GP and 1 wt% SP were added to 99 wt% PAO4 oil. Fluid mixture was then stirred for 10 min and sonicated for 30 min, resulting in a stable dispersion of GP oil. The dispersing performance is shown in Fig 4. The 0.01 wt% GP concentration was exceedingly difficult to assess. Hence, 0.05 wt% GP oil was prepared for comparison. After three days (Fig 4b), fluid **II** is darker and more turbid than fluid **I**. This finding indicates that SP can improve GP dispersion and stability in oil. The agglomeration phenomenon was also noted through careful observation. After nine days (Fig 4c), a considerable amount of precipitate was observed in each solution. In bottle **II**, some agglomeration was observed, exhibiting a deeper color than those of the other bottles. This finding further confirms that SP can effectively enable GP dispersion in the base oil.

After wear testing, the worn ball-and-plate specimens were cleaned with acetone and ethanol then dried. The morphologies were analyzed through optical microscopy (OLYMPUS BX50) and SEM (JSM-7001F; JEOL, Japan). The tribo-chemistry behavior was chartered by Raman spectroscopy and energy dispersive X-ray spectroscopy (EDAX-7760/68M). The wear volume was measured by Nano map (Aep), and the wear rate was calculated.

## Results and Discussion

### Tribological properties

**Fig 5:**
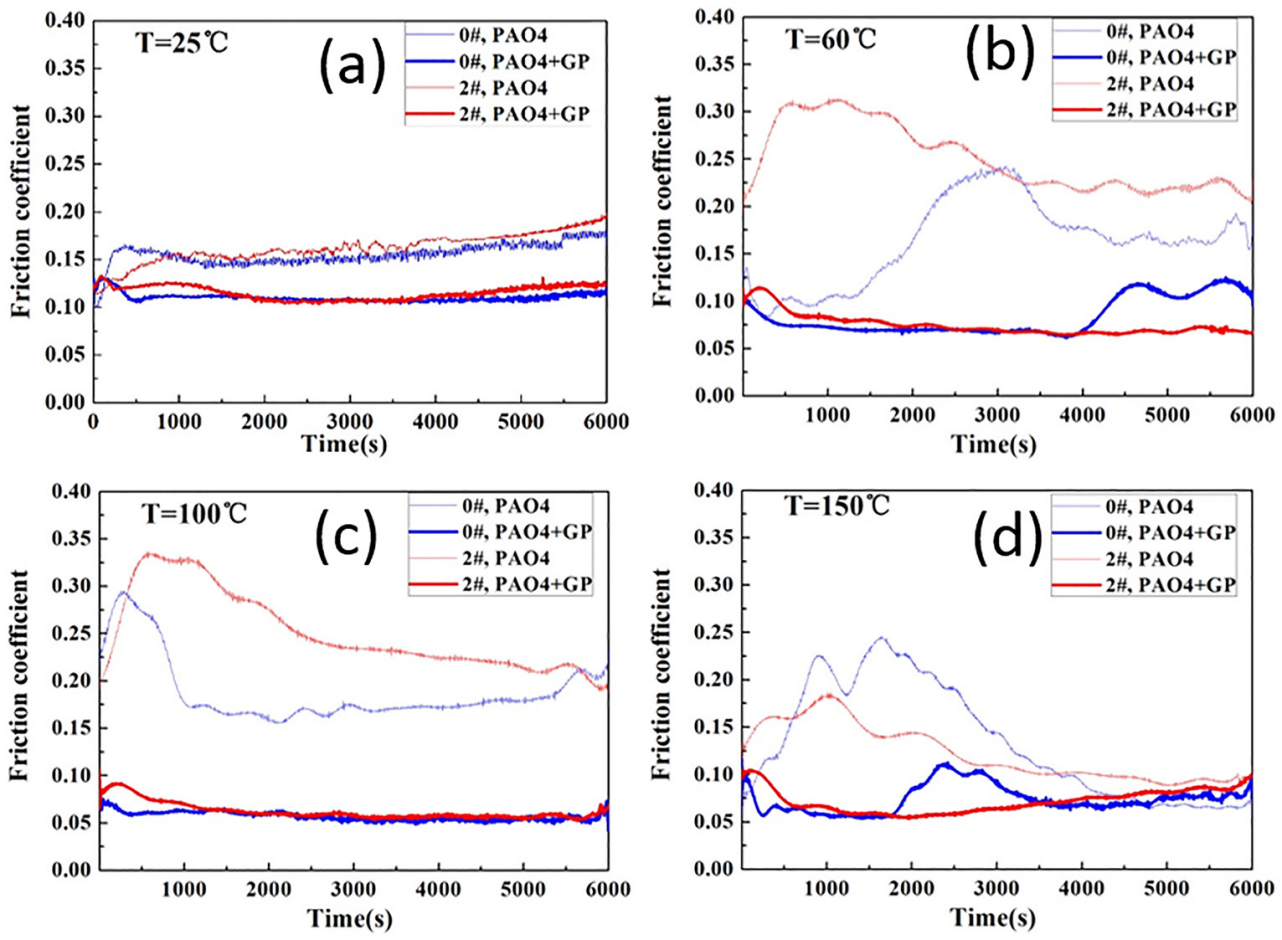
Friction coefficients at different temperatures.

The friction coefficients obtained from the tests on the textured and untextured plates differed significantly from each other (Fig 5). GP addition effectively reduced friction, and the coefficient of friction (COF) decreased by nearly 75%, especially at 60 and 100°C, relative to that of the oil. At room temperature, the COF was relatively stable because of sufficient oil thickness and mild oxidation reaction. In the initial stage at 150°C, GP effectively reduced COF, but the effect became less noticeable with the progress of time. The COF curves of the PAO4 oil present a rise and fall in the initial stage at temperatures 60, 100, and 150°C. This finding might be due to the mechanical removal action in the running-in period. The COF at 150°C was lower than those at 60 and 100°C, which may be attributed to the debris formed at high temperatures that can reach the interface and reduce wear.

The surface texture significantly influenced the COFs under pure PAO lubrication (Fig 5). Below 100°C, the COFs obtained from testing the dimpled plate exhibited a sharp increase at the beginning of the test, followed by a steady decrease after reaching a peak value.

Compared with the PAO4 base oil, the COF curves after GP oil addition were smaller and more stable under all the test temperatures. This finding may be due to the GP adsorbed on the tribo-surface and formed a protective film that separated the samples under contact preventing the occurrence of oxidation reaction [19–22]. The wear mechanism will be analyzed and discussed in the subsequent section.

**Fig 6:**
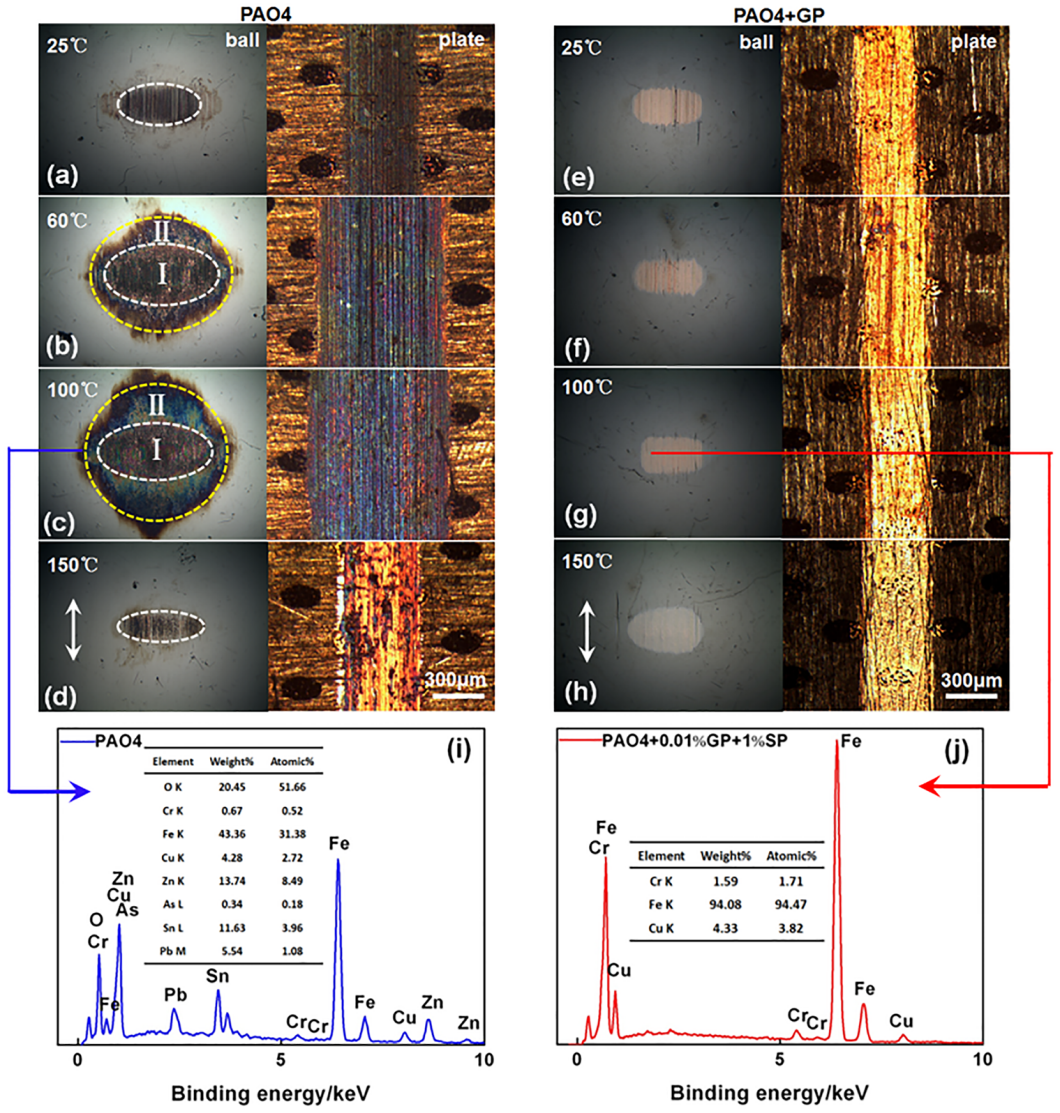
Morphology of the wear scars on the steel balls and 2# textured plates (a–h), as well as the EDS patterns of the wear scar on the ball (i, j).

The images of the wear scars are displayed in Fig 6. Lubricated by pure PAO4, the worn dimensions of the balls and plates dramatically varied with changing temperatures. The scar sizes of the balls and widths of the plates were smaller at 25 and 150°C and larger at 60 and 100°C. Especially, the wear scars of the balls were divided into two parts at 60 and 100°C, namely, the worn part **I** (area inside the white line) and the oxidized part **II** (intermediate area between the white and yellow line), respectively. Under GP-based oil lubrication, the wear scar was reduced relative to that under pure oil lubrication (Fig 6e–6h). Energy dispersive spectroscopy (EDS) analyses were carried out to estimate the chemical changes induced by friction. As shown in Fig 6i, except for the substantial oxygen content, many elements in the plate can be found in the worn ball. These elements include tin, zinc, and lead (correspond to the worn surface in Fig 6c). When the GP was added to the oil, the worn surface attained a bright color unlike that of the dark scar lubricated by pure PAO oil. The EDS result indicates the elemental transfer and that oxidation did not occur on the worn ball (Fig 6j). Interestingly, this phenomenon proves that the additive can inhibit the oxidation reaction effectively and modify the friction interface.

**Fig 7:**
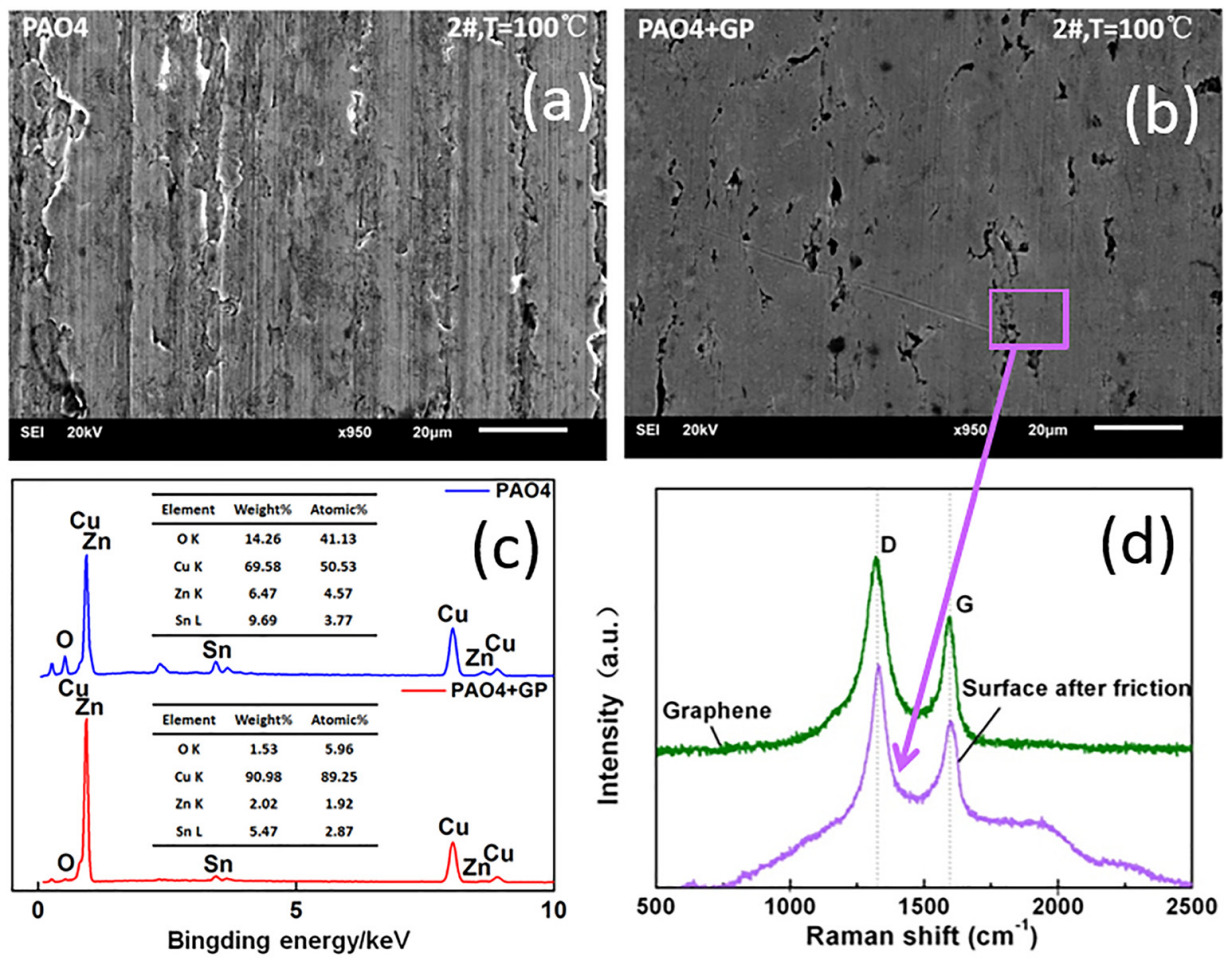
SEM and EDS of the worn surfaces. SEM images of the worn surfaces with (a) PAO4 oil and (b) PAO4 oil with GP; (c)EDS patterns and (d)Raman spectrum of the wear scar.

The SEM morphologies and EDS results of the worn plate surface are shown in Fig 7. Under the pure PAO4 oil, ploughing and wear debris could be observed (Fig 7). However, the wear scar was relatively smooth and shallow under the addition of GP in oil (Fig 7b). The EDS data revealed that the oxygen of the worn surface was higher under pure PAO4 oil, indicating the presence of oxidative wear phenomenon. The oxygen content of the worn surface was lower in the GP-based oil, revealing that GP can effectively inhibit the oxidation reaction to improve the tribological environment of the interfaces. Fig 7d displays the Raman spectra of the GP and the wear scar under the GP-containing oil. As observed, the D and G peaks of the worn surface were consistent with the peaks of GP. Therefore, GP possibly adsorbed on the bronze surface and formed a film during the wear process.

**Fig 8:**
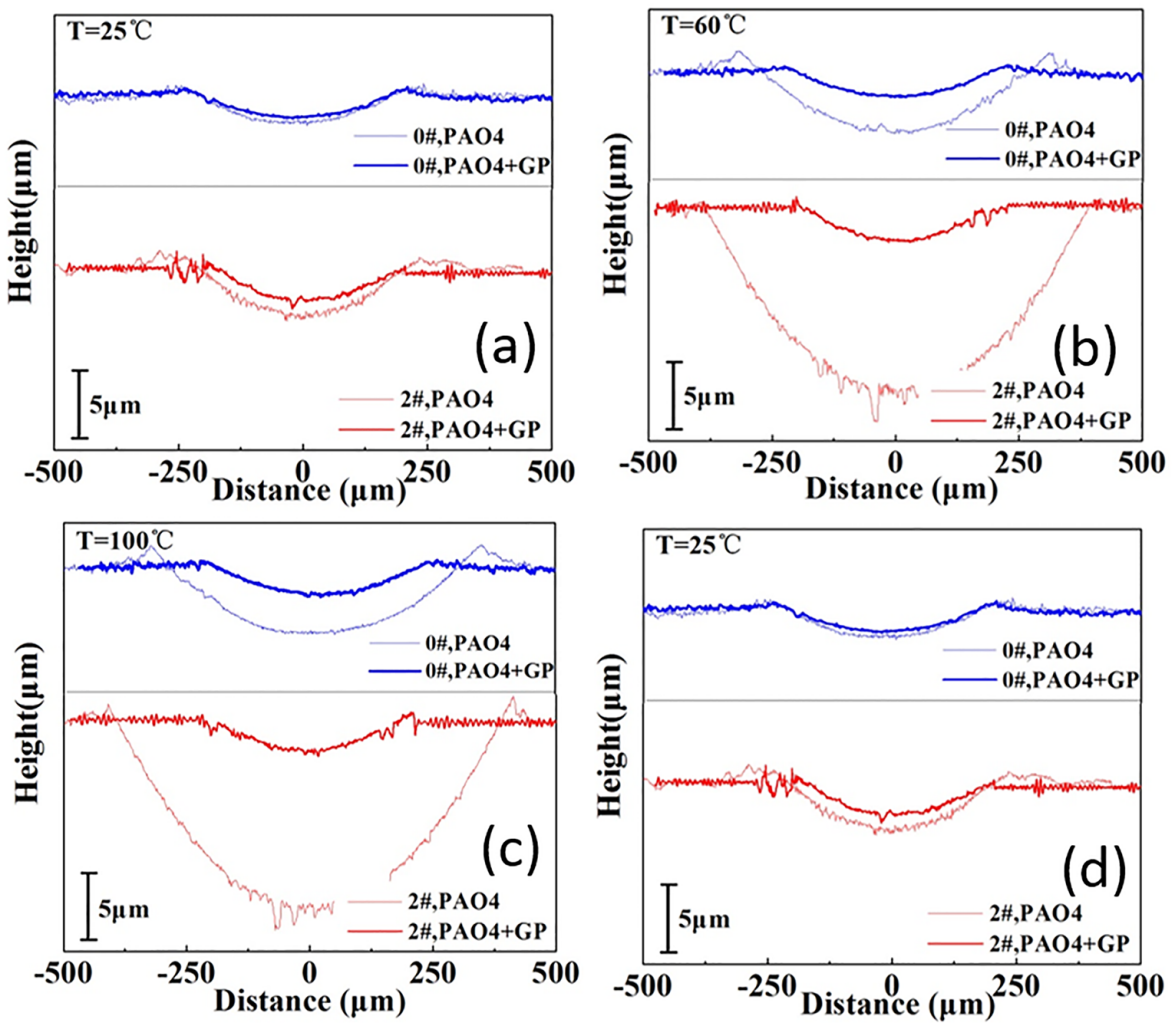
Cross-sectional profiles of the wear scars (samples 0# and 2#).

Fig 8 illustrates the cross-sectional profile of the wear scar. The wear scar profile lubricated by GP-containing oil was significantly shallower than that lubricated by pure PAO4. The distinction was evident at 60 and 100°C. After adding GP, the maximum depth of the wear scar reduced from 3.8 μm prior to addition to 2.6 μm after addition at 25°C (Fig 8a) and even from 14.2 μm to 2.7 μm at 60°C (Fig 8b).

### Effect of texture and wear mechanism

**Fig 9:**
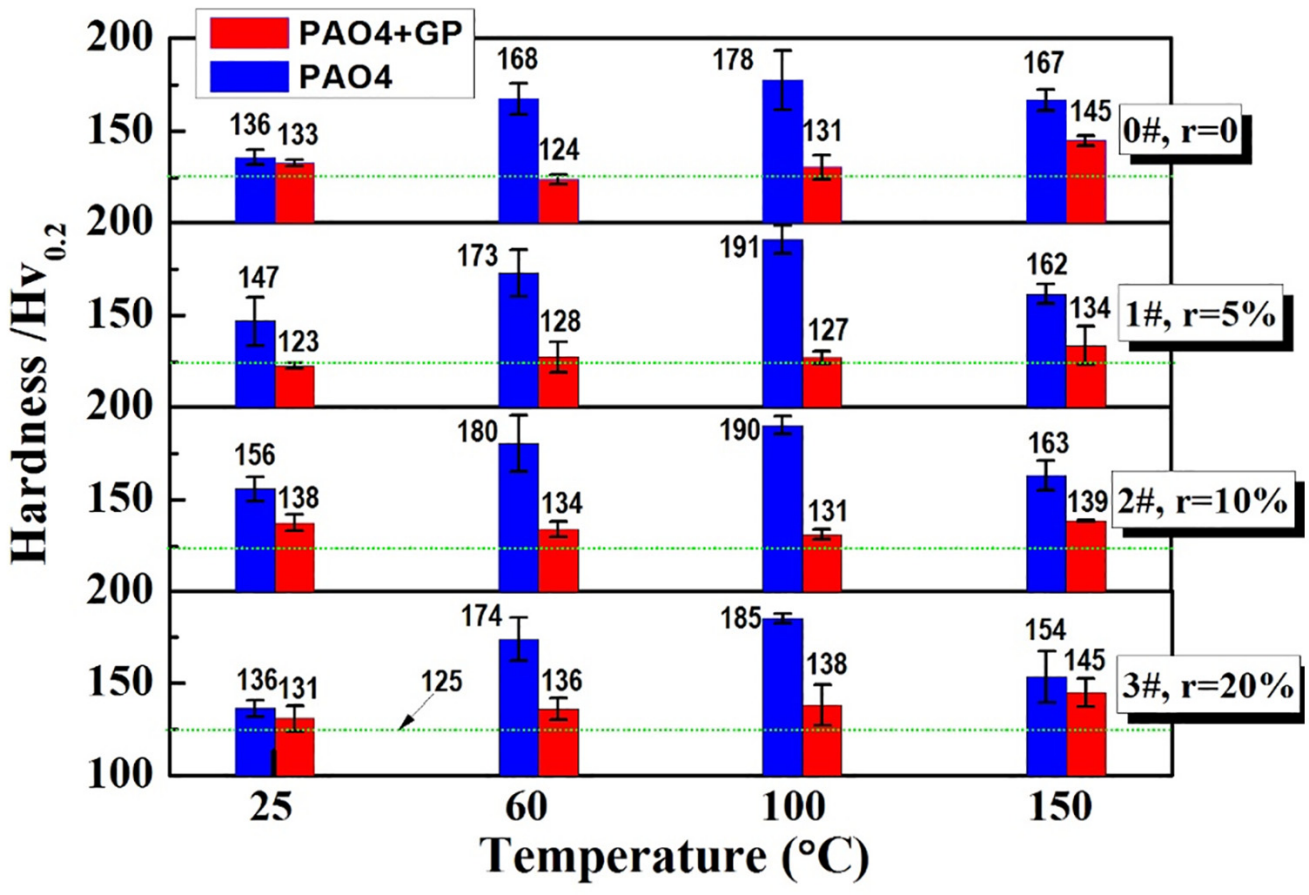
Hardness of the wear scar surface.

The hardness of the wear scars are presented in Fig 9. The hardness of the wear scar lubricated by the GP-containing oil was lower than that of pure PAO4 in all textured and non-textured samples, especially at 60 and 100°C. By comparing the hardness data, we conclude that the hardness remained constant regardless of temperature.

**Fig 10:**
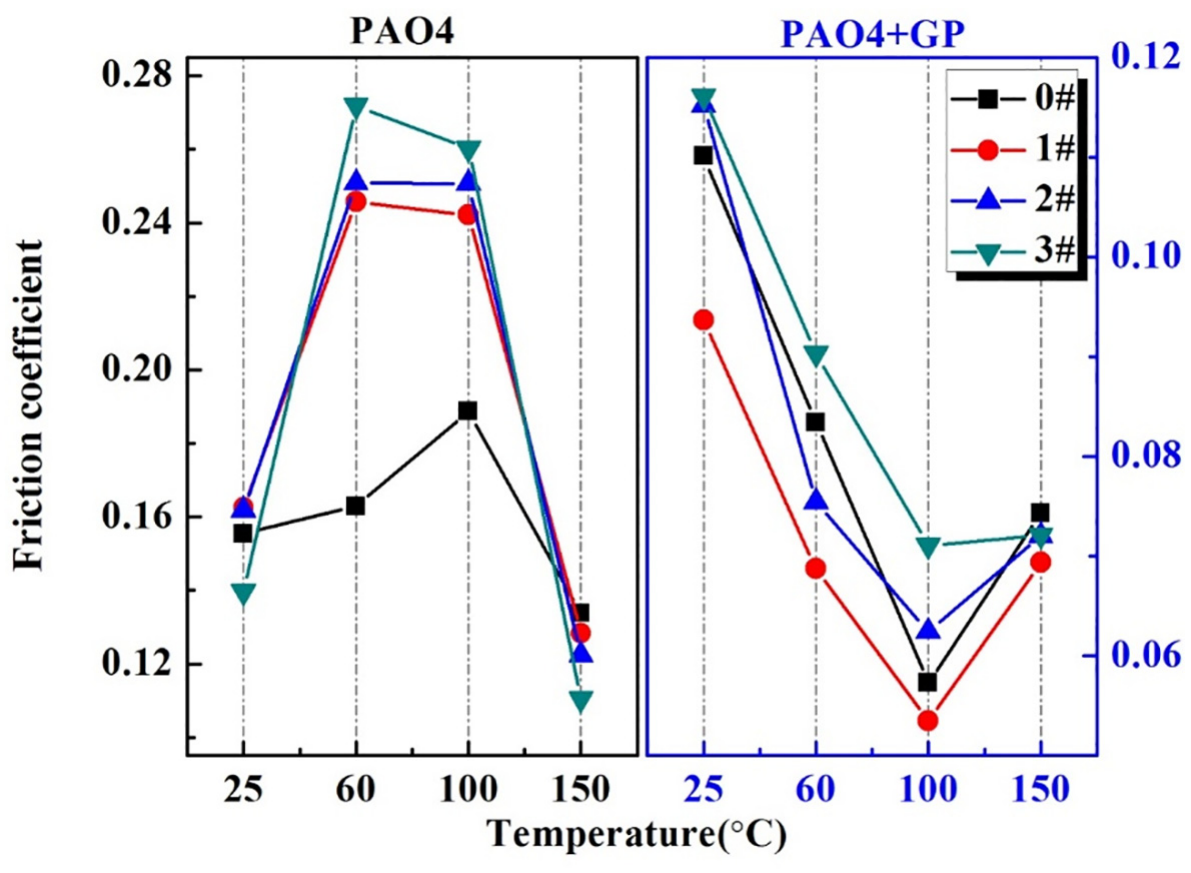
Comparison of the average COFs among different textured surfaces.

Fig 10 reveals the average COF values under different textured samples. Under pure PAO4 oil, the COFs exhibited similar tendencies with increasing temperature; the COFs rose at 60–100°C and then declined at 150°C. When the ratio of the surface texture increased, the high textured ratio reduced the high COF values at 60–100°C. The emergence of this phenomenon might be due to the presence of the dimples collecting debris at 25 and 150°C that becomes saturated at 60 and 100°C because of excessive amounts of debris that cannot be collected efficiently. By contrast, under GP-based oil lubrication, the COFs appeared to remain constant regardless of the textural changes. By detailed analysis, we determined that the COF of the 20% textured surface is higher than that of the 5% textured surface. These findings may be explained by the ability of the dimples to collect debris during the friction process and the magnification of surface roughness by excessive macroscopic dimples. We determined the optimal ratio between the two components to be 5%, which also achieved the lowest COF in the test.

**Fig 11:**
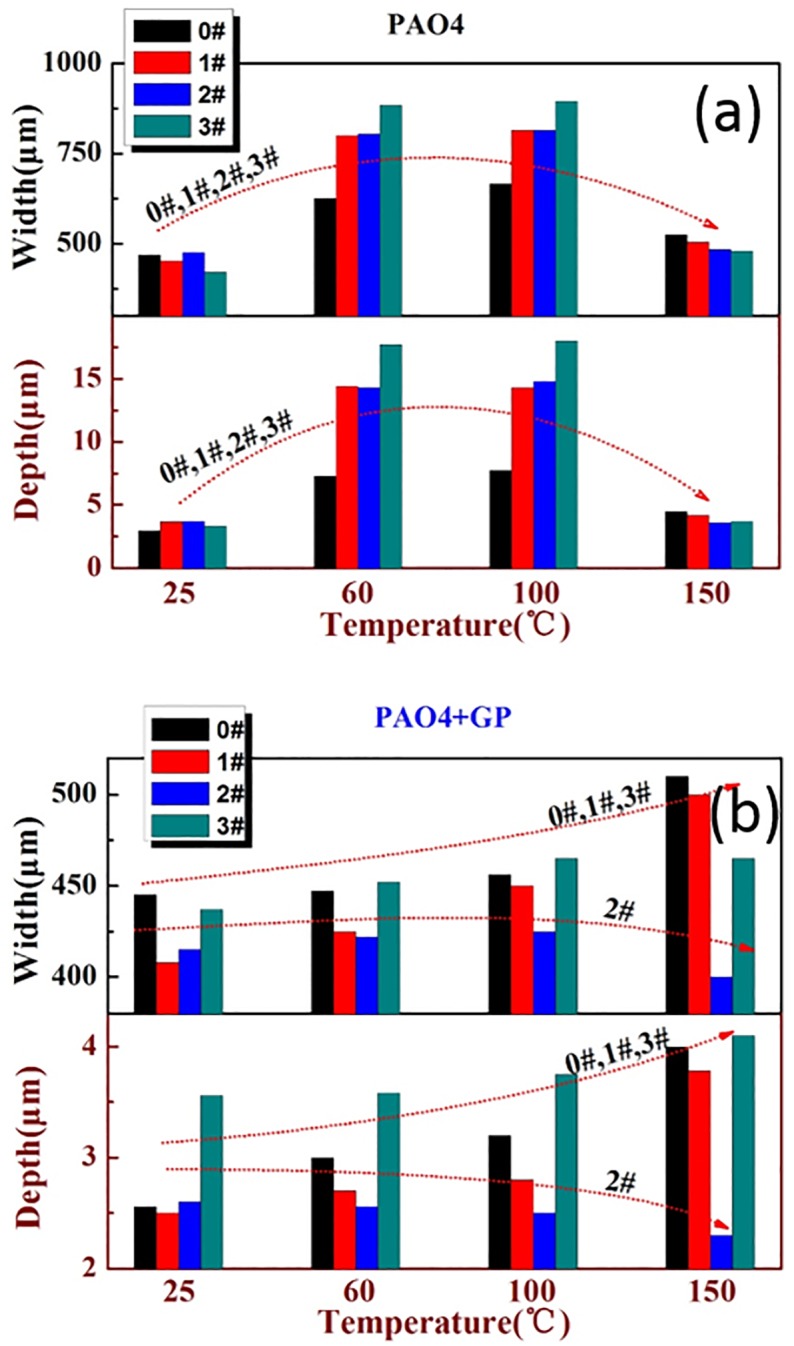
Widths and depths of the wear scars under different temperatures.

The depths and widths of the worn plate samples are depicted in Fig 11. Under pure PAO4 lubrication, the depths and widths of the wear scars under different textures exhibited the same trend with the change in temperature. In particular, the depths and widths increased initially and then decreased. The depths and widths were approximately the same at 25 and 150°C. At 60 and 150°C, the depths and widths increased with increasing texture ratio, whereas an opposite trend was observed at 25 and 100°C. Under the GP-based oil, the widths and depths of the wear scars of the samples exhibited an upward trend at texture ratios of 0%, 5%, and 20%, and a downward trend at 10%.

**Fig 12:**
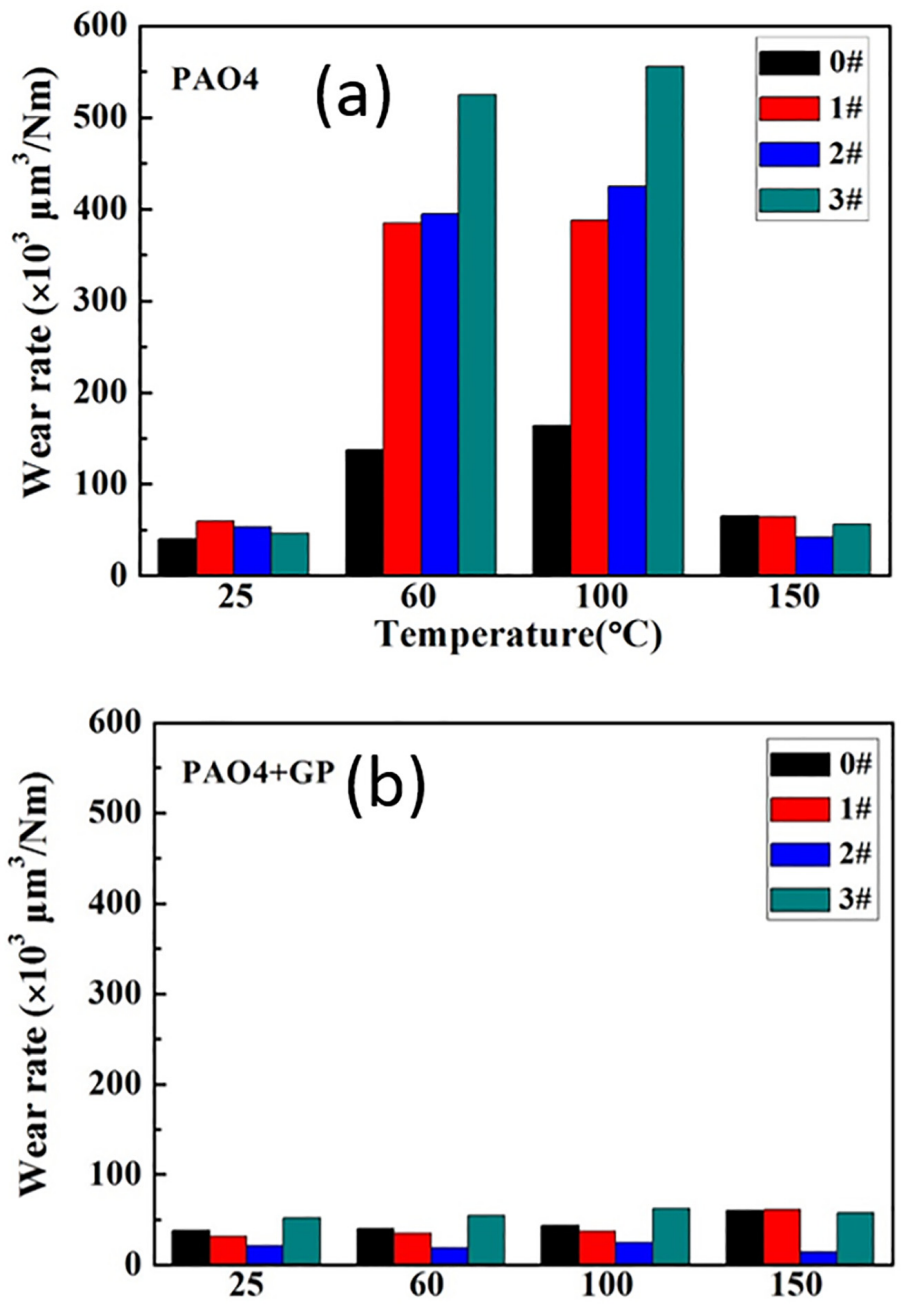
Wear rates under different temperatures.

The wear rates are shown in Fig 12. The four different textured surfaces exhibited similar trends after GP addition. At 60 and 100°C, wear rates dropped significantly, even up to 95% reduction, under GP-based oil lubrication. Furthermore, under this lubrication, the wear rate of the 10% textured surface is lower than those of the others at any of the temperatures tested. The temperature can affected the tribology situation during the tribology [23], J. Taha-Tijerina reported the Temperature-dependent viscosity variation of nanofluids- two dimensional (2D) atomic sheets, such as hexagonal boron nitride (h-BN) and graphene (GP)[24].

**Fig 13:**
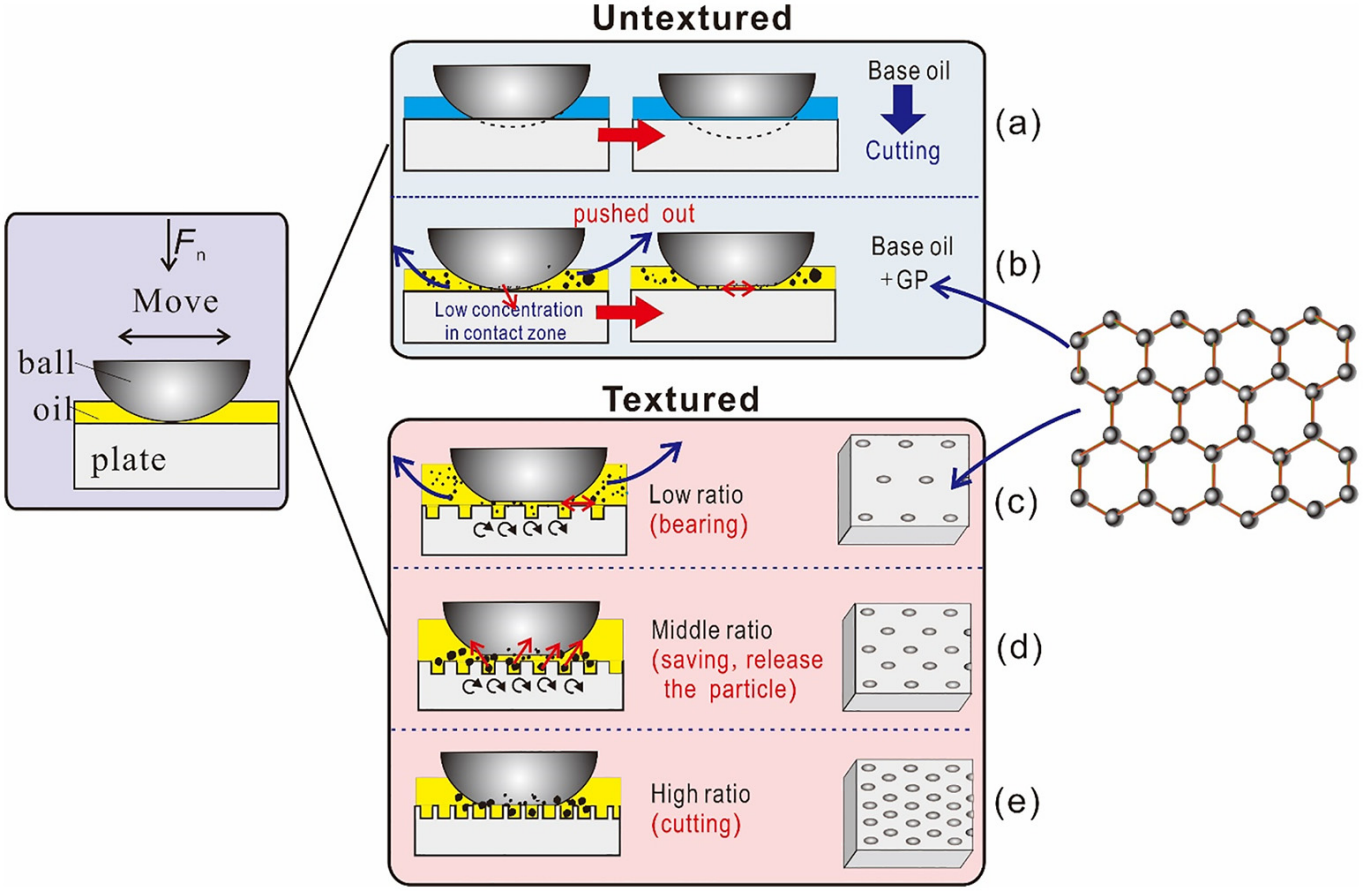
Schematic of the mechanism of the role of the GP additive in the texture plate.

As shown by the tests on tribological performance, GP-containing oil exhibited excellent anti-friction and anti-wear effects. GP possesses a lamellar structure and involves sliding between layers. This structure can then contribute to the anti-wear and anti-friction properties during tribology processing Understanding the lubrication mechanisms and anti-wear functions of additives is highly important. Fig 13 shows the wear mechanism of the GP additive on the textured plate. Forming stable GP dispersion in solution is difficult to achieve. A base oil with well-dispersed additives plays an important role in friction reduction and anti-wear action. In this research, a slow sliding speed of 5 mm was employed and hence corresponds to a boundary lubrication situation. Additionally, the point of contact in the ball-against-plate contact model involves high contact stress. In this case, the spherical cap can be easily cut off at the base oil (Figs 8 and 13a). The contact zone is small (<1 mm diameter) in the untextured plate. After GP addition, the particle hardly exists at the smooth tribological interface during the sliding process (Fig 13b). In the textured surface, the holes can store several particles and release and improve the contact state of the friction interface (bearing or formation of protective film) (Fig 13c–13e). However, exceedingly dense textures will cause the rubbing surface to be excessively rough, resulting in increased wear loss (Fig 13e). The area ratio of the texture should neither be exceedingly large nor exceedingly small. Raman spectroscopy determined the presence of GP on the wear scars after ultrasonic washing (Fig 8d). Two scenarios may explain this finding First, (a) GP may have adsorbed on the wear surface and formed a tribofilm. Second, (b) GP and lose materials may have collectively formed the debris and protected the interface. The interlayer friction behavior is affected by the GP structure, such as stacking form, relative sliding direction, size, defect, and number of layers [25–26]. However, many other forms of mechanisms exist and further research is needed to understand these processes better.

## Conclusion

Graphene(GP) has attracted much interest because of its excellent properties, such as mechanical strength and good conductivity. Its lamellar structure affords the material its potential for tribological applications. From the series of tests conducted, the following conclusions were drawn:

1. Span-80 generates a stable GP dispersion in PAO4 base oil by inhibiting the occurrence of the agglomeration phenomenon.
2. GP can effectively improve the anti-wear properties of contact surfaces. This improvement is most apparent at 60 and 100°C. The maximum COF can be reduced by 78%, whereas the maximum wear rate can be decreased by 90%.
3. Without GP additive, textured surfaces can exhibit increased wear losses at temperatures below 100°C. With the joint action of added GP and texture, the textured surface with an area ratio of 10% exhibits the best anti-wear performance among the different ratios under all the temperatures tested.

## Supporting Information

S1 File (RAR)
